# Associations of Alpha and Beta Interhemispheric EEG Coherences with Indices of Attentional Control and Academic Performance

**DOI:** 10.1155/2020/4672340

**Published:** 2020-02-05

**Authors:** Vasavi R. Gorantla, Sarah Tedesco, Merin Chandanathil, Sabyasachi Maity, Vernon Bond, Courtney Lewis, Richard M. Millis

**Affiliations:** ^1^Department of Basic Sciences, American University of Antigua College of Medicine, Antigua and Barbuda; ^2^Exercise and Nutritional Sciences Laboratory, Howard University Cancer Center and the Department of Human Performance and Leisure Studies, Washington DC 20060, USA; ^3^Department of Clinical Medicine, American University of Antigua College of Medicine, Antigua and Barbuda

## Abstract

*Introduction*. Heretofore, research on optimizing academic performance has suffered from an inability to translate what is known about an individual's learning behaviors to how effectively they are able to use the critical nodes and hubs in their cerebral cortex for learning. A previous study from our laboratory suggests that lower theta-beta ratios (TBRs) measured by EEG may be associated with higher academic performance in a medical school curriculum.

**Methods:**

In this study, we tested the hypothesis that TBR and academic performance may be correlated with EEG coherence, a measure of brain connectivity. We analyzed the interhemispheric coherences of the subjects involved in our prior study. TBR and coherence measurements were made at 19 scalp electrode recording sites and 171 electrode combinations with eyes open and closed (EO, EC). Control data were acquired during a session of acclimation to the research protocol 3 d before an initial examination in anatomy-physiology (control exam) and were repeated five weeks later, 3 d before a second exam covering different anatomy-physiology topics (comparison exam).

**Results:**

Between the control and comparison exams, beta coherences increased significantly at the frontal pole, frontal, parietal, midtemporal, posterior temporal, and occipital recording sites under the EO condition and at the inferior frontal, central, midtemporal, and posterior temporal sites under the EC condition. Alpha coherences increased significantly at the same sites and under the same EO/EC conditions as found for the beta coherences. The beta coherences were negatively correlated with the TBR and were positively correlated with the comparison exam score at the midfrontal electrode site (F3-F4) but only under the EO condition. Beta and alpha coherences at the midfrontal, inferior frontal midtemporal, posterior temporal, and occipital sites were also negatively correlated with the average TBR under the EO condition.

**Conclusions:**

Lower TBR, an indicator of attentional control, was associated with higher alpha and beta interhemispheric coherences measured with eyes open at sites overlying the frontal, temporal, and occipital cortices. Changes in EEG coherences and TBRs might be useful as neurophysiological measures of neuroplasticity and the efficacy of strategies for preventing academic underachievement and treatments for improving academic performance.

## 1. Introduction

Interhemispheric EEG coherence between scalp electrodes placed at symmetrical sites overlying the left and right cerebral hemispheres is a measure of their connectivity to a common neural network [1]. The advent of quantitative electroencephalography (qEEG) has made it common practice to measure interhemispheric coherences. For the standard frequency ranges of delta (0-3 Hz), theta (4-7 Hz), alpha (8-12 Hz), and beta (13-30 Hz) at 19 standard electrode recording sites, coherence measurements yield data for 171 electrode combinations [2]. Coherence is essentially a measure of cospectral in-phase and quadspectral out-of-phase relationships between the various recording sites, thereby measuring the amount of phase-locking between functionally connected sites [2]. Frequency-specific functional connectivity appears to reflect the functions of the network nodes and hubs ascribed to Brodmann's areas of the cerebral cortex. For example, frontal delta coherence is reported to reflect the amount of communication between distant brain sites during decision-making [3]. Delta (low) frequency oscillations, observed mainly during deep slow-wave-sleep and meditation, are also well-suited for relatively long-distance communication during wakefulness, and the frontal lobes are shown to support executive functions such as decision-making. Theta waves, seen primarily at the onset of sleep, are shown to emanate from the thalamus and hippocampus during wakefulness [4, 5]. Frontal theta activity is maximized during declarative and navigational memory usage [4, 5].

It is generally observed that in healthy humans, alpha amplitudes increase and low beta (beta frequencies close to alpha, 12-15 Hz) amplitudes decrease when the eyes are closed and *vice versa* when eyes are opened [6]. This type of alpha suppression is known as “alpha blocking” and does not appear to result from visual stimulation because it occurs in the absence of light, in a completely dark environment [7]. This behavioral response is also referred to as event-related desynchronization because it is seen in response to various tasks and is therefore likely to be a reflection of cerebral cortical activation or cortical excitation [8]. Alpha waves are thought to represent the brain's default mode network, optimized under eyes-closed conditions [9, 10]. Frontal alpha coherence appears to reflect the amount of task-related vigilance and attention [9, 10]. Beta waves increase under eyes-open conditions, and frontal beta coherence seems to indicate the degree of mental effort involved in performing problem-solving tasks [10, 11]. A previous report from our laboratory demonstrates that increases in the topographically averaged theta and beta voltages increase and theta/beta voltage ratios (TBR) decrease during the academic challenge presented to first-semester students preparing for their first two medical school examinations [12]. In the same study, the TBRs, an index of attentional control, were negatively correlated with the second examination scores, the first examination score serving as the control. These findings motivate the hypotheses of the present study that the frontal theta and beta coherences would be negatively correlated with the TBRs and positively correlated with the second examination scores, thereby providing novel biomarkers for academic underachievement and the efficacy of therapeutic strategies for improving academic performance.

## 2. Methods

This study was approved by the American University of Antigua College of Medicine Institutional Review Board. The subjects were ten healthy first-semester male medical students recruited at the time of their matriculation. Nine of the ten subjects completed the study. Each subject's qEEG was performed twice, 3 d before each of their first two block examinations in anatomy-physiology. The measurements made 3 d before the first exam served as a baseline control and acclimation to the laboratory and experimental protocol. The measurements made 3 d before the second exam served as comparison data to test the hypotheses that the frontal theta and beta coherences measured 3 d before the second exam would be greater than the baseline control values, would be negatively correlated with the TBRs measured at the same time, and would be positively correlated with the second exam scores.

The measurement protocols are described in detail in our previous report [12]. Briefly, qEEG measurements were performed using the Brain Master Discovery system (Bedford, Ohio, USA) at the same time of day. The subjects were instructed to refrain from the use of medicines, caffeine, and alcohol for 24 h prior. qEEG measurements were made for 10 consecutive minutes using a well-fitted electrode skull cap employing 19 recording sites with spacing based on the international 10/20 system. Subjects were seated upright, in a darkened room, with both feet on the floor with eyes fixated on a dot marked on a blank white wall under an eyes-open condition and in the same position under a volitional eyes-closed condition.

The raw qEEG recording was subjected to manual artifact removal. Coherences were computed for each of four frequency bands (delta 0-3 Hz, theta 4-7 Hz, alpha 8-12 Hz, and beta 13-30 Hz) for each symmetrically placed electrode pairs as follows: frontal pole (Fp1-Fp2), frontal (F3-F4), inferior frontal (F7-F8), central (C3-C4), parietal (P3-P4), midtemporal (T3-T4), posterior temporal (T5-T6), and occipital (O1-O2).

Statistical significance of differences in the mean delta, theta, alpha, and beta coherences between the baseline qEEG control measurements made 3 d before the first exam (control) and those made 3 d before the second comparison exam was determined by one-way ANOVA with post hoc *t*-testing. Theta/beta ratios (TBRs) were computed from the mean voltage magnitudes in the theta and beta bandwidths. Significance of correlations between the eyes-open and eyes-closed qEEG coherences, TBR, and exam scores was determined by linear regression analysis with reporting of the Pearson product-moment correlation coefficient. Significance was guaranteed at *P* < 0.05.

## 3. Results

### 3.1. Frontal Increases in Coherences at Fp1-Fp2, F3-F4, and F7-F8

**Figure 1:**
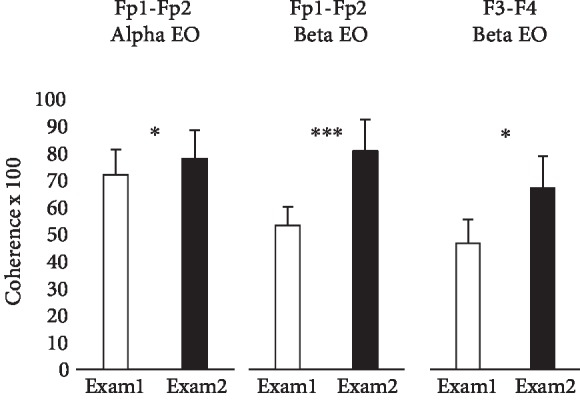
Frontal pole and midfrontal EEG coherences. Bars show means ± standard errors of alpha and beta coherences measured from the scalp overlying the frontal pole (Fp1-Fp2) and beta coherence measured from the scalp overlying the midfrontal cortex (F3-F4), under eyes-open (EO) conditions. Subjects were 9 healthy male medical students studied 3 d before each of their first two block exams in an integrated anatomy-physiology course (Exam 1, Exam 2). ∗ indicates differences significant at *P* < 0.05, and ∗∗∗ indicates differences significant at *P* < 0.001.

**Figure 2:**
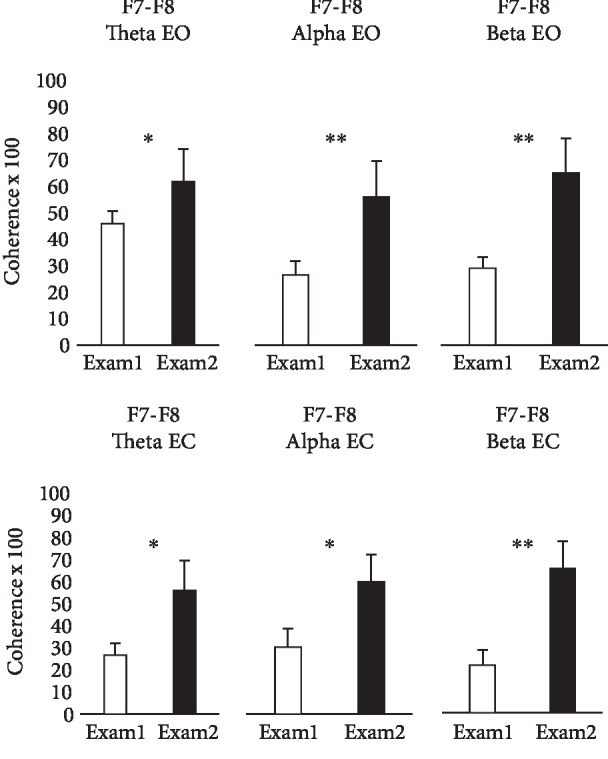
Inferior frontal EEG coherences. Bars show means ± standard errors of theta, alpha, and beta coherences measured from the scalp overlying the inferior frontal cortex (F7-F8), under eyes-open (EO) and eyes-closed (EC) conditions. Subjects were 9 healthy male medical students studied 3 d before each of their first two block exams in an integrated anatomy-physiology course (Exam 1, Exam 2). ∗ indicates differences significant at *P* < 0.05, and ∗∗ indicates differences significant at *P* < 0.01.

Figures 1 and 2 present the changes in frontal EEG coherences between the baseline control measurement 3 d before each subject's first block exam and 3 d before their second block exam.  Figure 1 shows that significant increases in alpha and beta coherences were found at electrode pair Fp1-Fp2 overlying the frontal poles and an increase in beta coherence at F3-F4 overlying the superior frontal lobes, only under the eyes-open condition.  Figure 2 shows that there were significant increases in theta, alpha, and beta coherences at F7-F8 overlying the inferior frontal lobes with eyes open, as well as with eyes closed.

### 3.2. Decreases in Delta Coherence at C3-C4

Decreases in delta bandwidth coherences were found at C3-C4 overlying the central sulcus under both eyes-open and eyes-closed conditions. Significant decreases in coherences were not observed in the theta, alpha, and beta frequencies.

### 3.3. Increases in Theta Coherences at C3-C4 and T3-T4

In addition to the aforementioned increase in theta coherence at F7-F8 shown in Figure 2, there were increases in theta coherences at C3-C4 and at T3-T4, the latter overlying the midtemporal lobes, with eyes closed.

### 3.4. Increases in Alpha Coherences at T3-T4 and T5-T6

In addition to the increases in alpha coherences found at Fp1-Fp2 and F7-F8 shown in Figures 1 and 2, there were increases in alpha coherences at T3-T4 and T5-T6, the latter overlying the post temporal lobes, under both the eyes-open and eyes-closed conditions.

### 3.5. Increases in Beta Coherences at C3-C4, T3-T4, T5-T6, P3-P4, and O1-O2

In addition to the increases in beta coherences at Fp1-Fp2, F3-F4, and F7-F8 shown in Figures 1 and 2, increases in beta coherences were seen at P3-P4 overlying the parietal lobes and at O1-O2 overlying the occipital lobes, only under the eyes-open condition. Increased beta coherence was found at C3-C4 under the eyes-closed condition and at T3-T4 and T5-T6 under both the eyes-open and eyes-closed conditions.

Between the control and comparison exams, beta coherences increased significantly at the frontal pole, frontal, parietal, midtemporal, posterior temporal, and occipital sites under eyes-open conditions and at the inferior frontal, central, midtemporal, and posterior temporal sites under eyes-closed conditions. Alpha coherences increased significantly at the same sites and under the same conditions as found for the beta coherences, except at the central and occipital sites where the increases were not significant.

### 3.6. Significant Correlations

Across all bandwidths, the eyes-open voltage amplitudes and coherences were positively correlated with the eyes-closed voltage amplitudes and coherences and the voltage amplitudes were positively correlated with the coherences (*r* = 0.86‐0.97, *P* < 0.001).

The alpha and beta coherences were negatively correlated with the average TBR, at Fp1-Fp2, F3-F4, F7-F8, T3-T4, T5-T6, and O1-O2 only under the eyes-open condition (*r* = −0.60 to *r* = −0.80, *P* = 0.05 to *P* = 0.01). Neither alpha nor beta coherences were significantly correlated with TBR at P3-P4.

The alpha coherences were not significantly correlated with the second exam score.

**Figure 3:**
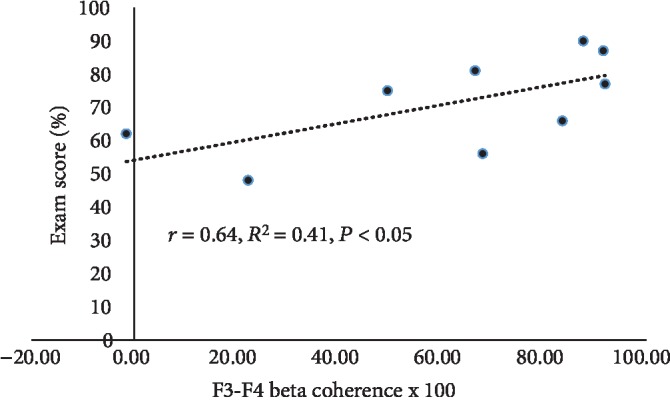
Association of midfrontal beta coherence and exam score. Linear regression analysis and line of best fit demonstrating significant correlation between the beta coherence measured from the scalp overlying the midfrontal cortex and the second exam score. Subjects were 9 healthy male medical students studied 3 d before each of their first two block exams in an integrated anatomy-physiology course (Exam 1, Exam 2).

Figure 3 shows that the beta coherences measured 3 d before the second (comparison) exam were positively correlated with the second exam score, only at F3-F4 under the eyes-open condition.

## 4. Discussion

The main finding of this study is that, under eyes-open conditions, the frontal beta coherences measured before a second (comparison) medical school anatomy-physiology exam were negatively correlated with the average theta-beta ratios (TBRs) measured at the same time. The frontal beta coherences were also positively correlated with the percent correct score on the second exam, administered 3 d after the qEEG measurements.

External conditions for conducting the two exams were not different. For example, although the specific questions and topics were different for the two exams, they were both conducted online, in the same computer lab-classroom, using the same computers and seating arrangement. This environmental constancy raises the question: what factors changed just before the second exam to make the correlations significant that were not significant just before the first exam? The simplest answer is that there were improvements in frontal connectivity during wakefulness and attentional control, evidenced by a significant negative correlation between TBR and frontal beta coherence. It is beyond the scope of the present study to determine the specific training-related factors which explain the aforementioned change in significance. Similar TBR-beta wave correlations are reported in a large mixed-gender cohort of university students following a gambling task with changing reward-punishment contingencies which were interpreted as an improvement in behavioral flexibility [13]. We believe that the gambling paradigm is an apt one for our study on medical students adapting to life in a different country and preparing for their first two high-stakes medical school exams. Attending medical school at the cost of approximately $60,000 USD per annum in the Caribbean is likely to be a high-risk gamble for the U.S. and Canadian students making up the majority of our student body. Rigorous daily basic science lectures and small group learning activities combined with the requirement to integrate the basic science information with clinical medical applications is a daunting task, especially when a passing score of 70% needs to be maintained, based on only four high-stakes exams, two per semester. These facts suggest that the main findings of our study, negative correlation between TBR and frontal beta coherence in conjunction with positive correlation between frontal beta coherence and second comparison exam score, are likely to represent biomarkers for a training-related effect. Such an effect is, no doubt, required for successful adaption to the reward-punishment environment of medical school by adjusting one's lifestyle and study habits for increased cerebral cortical attentional control and behavioral flexibility.

The significant negative correlations between beta bandwidth coherences and the average TBR were found at virtually all sites studied under eyes-open conditions, except for the parietal P3-P4 electrode pair where the correlations were not significant. The correlations between beta coherences and academic performance were restricted to the scalp overlying the midfrontal lobes with eyes open, which were not found in other regions of the scalp or with eyes closed. The frontal lobes are known to be hubs for the neural networks supporting thinking, planning, executive functions, mood control, and motor execution [8, 14]. Our subjects were seated and, with eyes open, were instructed to stare at a mark on a blank white wall for ten minutes while recording their EEGs. The amplitudes of beta waves are known to be minimized with eyes closed due to the alpha-blocking response wherein generation of alpha waves appears to inhibit generation of beta waves. Beta waves are, therefore, maximized with eyes open. This fact is consistent with our finding that the aforementioned correlations were observed only under eyes-open conditions and provides guidance for future research that might attempt to investigate these relationships under eyes-closed conditions. It is correct practice to compare qEEG recordings under eyes-open with those under eyes-closed conditions in the same subjects precisely because the brain's neural networks appear to be highly sensitive to the visual information added with eyes open. Indeed, it is estimated that 90% of the information a normal healthy person learns is dependent on visual input and more than 80% of the external input to the brain is processed by visual pathways [15].

Across the four wavelengths studied, coherences either increased significantly or did not change, but did not decrease at any of the brain sites. Low and high EEG coherences have been reported for a variety of neurological and neuropsychiatric disorders including autism, Alzheimer's, Parkinson's, and schizophrenia [1]. Whether coherence is high or low seems to vary with the particular site and disorder. Hypocoherence is thought to reflect disruption of the brain's white matter axon pathways connecting the two distinct regions underlying the particular scalp electrodes, and hypercoherence may reflect compensation for various metabolic states such as low voltage amplitude or low dominant frequency within a particular bandwidth [16]. Our findings of significant site- and frequency-specific increases in interhemispheric coherences between the control and comparison measurements five weeks apart suggest that the academic challenge of medical school may force the left and right cerebral hemispheres to increase their connectivity and communication, a key aspect of neural plasticity.

Increases in delta bandwidth coherences were found at C3-C4 overlying the central sulcus and the anterior cingulate gyrus under both the eyes-open and eyes-closed conditions. C3-C4 is a hub for volitional movement, attention, and long-term memory functions [17]. During wakefulness, delta power is thought to reflect relatively long-distance communication between cortical regions [18]. High delta power and coherence have been reported during meditation and in other physiological states associated with lower levels of consciousness [19]. Evidence is emerging that delta coherence also reflects communication between areas of the ventral tegmental area, the brain's main reward pathway [3]. In the present study, the medical student subjects were seated at rest either with eyes closed or with eyes open staring at a wall, in a darkened room. It is common for medical students to self-report sleep deprivation. Therefore, our finding with respect to increased delta coherence at C3-C4 may reflect an increased capacity for control of consciousness. It is also plausible that a perception of reward is associated with lower levels of consciousness in medical students taking a break from intense studying.

The increase in theta coherences observed at F7-F8 under both the eyes-open and eyes-closed conditions is likely to reflect an increase in functionality associated with Broca's area in the inferior frontal lobes. Low theta and gamma coherences are reported in patients with Broca's aphasia wherein both phonological and working memory processes are, in combination, dysfunctional [20]. The increase in theta coherence at C3-C4 under the eyes-closed condition is also likely to reflect increased functionality of working memory associated with control of attentional and reward behaviors as is shown in rodent brain training studies targeting the anterior cingulate cortex [21]. The increase in theta coherence at T3-T4 overlying the midtemporal lobes under the eyes-closed condition is likely to reflect increased functionality of language, auditory perceptions, long-term memory, and/or emotional control. The hippocampus plays an important role in all the aforementioned functions and is also a primary source of theta oscillations [22]. Those increases in theta coherences at C3-C4 and T3-T4 were only observed under eyes-closed conditions which is inconsistent with our finding that voltage amplitudes and coherences were highly correlated positively and with a report that there were no significant differences in theta power at these sites in healthy young adult undergraduate engineering and science students. The finding in our healthy adult medical students seems to be, nevertheless, logical based on reports that theta activity is enhanced during lower levels of consciousness, usually studied during eyes-closed conditions.

Increases in alpha coherences were found at Fp1-Fp2 overlying the frontal pole under the eyes-open condition. This finding is inconsistent with what is reported for healthy adult subjects about alpha power. Not only at the frontal pole, but throughout the brain, the normal physiological response to eye closure, known as “alpha blocking,” involves an increase in the voltage amplitude of alpha waves and decreases in amplitude of beta waves, concomitantly [8]. Hence, because of our aforementioned finding that voltage amplitudes were highly positively correlated, eye opening should have been associated with decreases in alpha power and coherence in all brain regions. The concomitant increases in alpha coherence at F7-F8, at T3-T4, and at T5-T6 overlying the post temporal lobes under both the eyes-open and eyes-closed conditions are consistent with what is known about connectivity within the arcuate fasciculus and its role in language processing [23, 24], thereby suggesting an academic challenge-induced increase in language functionality in our study subjects. Mastery of knowledge in medical school is, to a great extent, a process of listening and reading wherein technical material is presented in lecture and PowerPoint presentation formats requiring excellent auditory perception and reading comprehension. The added challenge at our institution is that we employ a diverse teaching faculty expressing a variety of accents that would also require our students to develop excellent auditory discrimination skills. This interpretation is consistent with a report that cortical alpha waves appear to be useful for solving the “cocktail party problem” of discriminating auditory noise from speech [25].

In the present study, increases in beta coherences were seen at Fp1-Fp2, F3-F4, and P3-P4 overlying the parietal lobes and at O1-O2 overlying the occipital lobes, only under the eyes-open condition. These findings are consistent with the alpha-blocking response wherein eyes-open conditions should maximize beta power and also with our finding that voltage amplitudes were highly positively correlated with coherence across all the bandwidths studied. Frontal beta coherence is shown to support control of the frontal eye fields [26], and the increases in Fp1-Fp2 and F3-F4 frontal beta coherences could, therefore, be indicative of increased oculomotor control in our subjects, an obvious skill enhancement that might be entrained by academic challenges requiring a lot of reading. Parietal beta activity at P3-P4 is shown to support attentional control and short-term memory functions [27]. The increase in P3-P4 beta coherence with eyes open which we observed is consistent with what would be predicted for an intense academic challenge requiring enhancement of attentional control and short-term memory functions. Occipital beta activity supports visual perception and spatial processing [28, 29]. The increases in beta coherence under the eyes-open condition in our subjects are, therefore, likely to reflect the increased functionality in visual processing required by an academic challenge such as medical school learning. Increases in beta coherences at F7-F8, at T3-T4, and at T5-T6 were observed under both eyes-open and eyes-closed conditions, therefore suggesting a robust finding roughly corresponding to the distributions of the arcuate fasciculus supporting language functions, as previously discussed for similar findings at the frontal poles, inferior frontal, and parietal cortices.

Although we found highly significant positive correlations between voltage amplitudes and coherence in each of the bandwidths studied, one should exercise caution in interpreting that such should always be the case. For example, it is reported that for mu waves, within the alpha bandwidth, voltage amplitudes, measured in fast Fourier transform (FFT) units of power, decrease during movement, as well as while watching the movement of others, in conjunction with an increase in coherence [30]. The mu response reflects the physiological underpinnings of the two measurements, voltage amplitude (or power) and coherence. Voltage amplitude, often expressed as power, measures the activity of dendrites of the multipolar pyramidal cells within the cerebral cortex which are firing synchronously at sites underlying each recording electrode at any given time. Coherence, on the other hand, measures the amount of phase-locking between two sites, in this study, between the same, symmetrical sites on the scalp overlying the left and right cortices. The symmetrical sites in the left and right hemispheres are known to possess the same macroscopic structure and are thought to share similar, but not precisely the same, functions. This concept is closely allied to that of lateralization of cerebral cortical functions [31]. Coherence reflects the amount of connectivity and communication between the cortices underlying two electrode recording sites.

## 5. Conclusions

We found that beta coherences measured 3 d before a comparison exam were negatively correlated with the site-averaged TBR and were positively correlated with the exam score at the midfrontal electrode sites (F3-F4) under eyes-open conditions. These findings are consistent with our previous report of positive correlation between voltage amplitude and coherence and with current knowledge about functions of the midfrontal lobes—thinking, planning, and decision-making. Heightened beta coherence associated with lowering of TBR may be a useful qEEG marker for high academic performance in students transitioning to medical school. Future studies should determine whether these findings apply to other types of academic challenges and cognitive performances.

## Data Availability

The data used to support the findings of this study are available from the corresponding author upon request.
